# HIV assisted partner services (aPS) to support integrated HIV and hypertension screening in Kenya: a pre-post intervention study

**DOI:** 10.1186/s12889-023-17205-2

**Published:** 2023-12-01

**Authors:** Beatrice Wamuti, Betsy Sambai, Christine Magambo, Margaret Ndegwa, Paul Macharia, Tecla M.Temu, Carey Farquhar, David Bukusi

**Affiliations:** 1 Harvard T. H. Chan School of Public Health, Harvard University, 677 Huntington Avenue, Boston, MA 02115 USA; 2 University of Washington - Kenya, Nairobi, Kenya; 3 Voluntary Counselling and Testing (VCT) and HIV Prevention Unit, Kenyatta National Hospital, Nairobi, Kenya; 4 Research and Programs Department, Kenyatta National Hospital, Nairobi, Kenya; 5 Department of Global Health, University of Washington, Seattle, USA; 6 Department of Epidemiology, University of Washington, Seattle, USA; 7 Department of Medicine, University of Washington, Seattle, USA

**Keywords:** HIV, Assisted partner services, Hypertension, Integration

## Abstract

**Background:**

People living with HIV (PLWH) have a higher risk of developing hypertension compared to HIV uninfected individuals. HIV assisted partner services (aPS), where PLWH are assisted by a healthcare provider to disclose their status to sexual and / or drug injecting partner(s), offers an opportunity for integrated HIV and hypertension screening. We evaluated the feasibility of the aPS model in supporting integrated HIV and hypertension screening at the Kenyatta National Hospital, Kenya.

**Methods:**

Between August 2019 and December 2020, we conducted a pre-post intervention study. We enrolled women receiving HIV testing services (HTS) with confirmed hypertension (female index clients) and traced their male relatives for HIV and hypertension screening and reviewed management at 3-months. Hypertension was defined as systolic blood pressure (SBP) ≥ 140 mmHg, diastolic blood pressure (DBP) ≥ 90 mmHg, and/or use of antihypertensive medication.

**Results:**

One hundred female index clients (median age: 55 years; interquartile range (IQR): 47–65) mentioned 165 male relatives (median: 49 years; IQR: 40–59) of whom 35% (*n* = 58/165) were enrolled. Of the male relatives, 29% had hypertension (*n* = 17/58), 34% had pre-hypertension (*n* = 20/58), and none were HIV-positive (*n* = 0/58). Among the female index clients, there was a statistically significant decline in SBP (pre: 156 mmHg, post: 133 mmHg, *p*-value: < 0.0001) and DBP (pre: 97 mmHg, post: 80 mmHg, *p*-value: < 0.0001), and increase in antihypertensive medication uptake (pre: 91%, *n* = 84/92; post: 98%, *n* = 90/92; *X*^2^: 4.3931, *p*-value: 0.036) relative to baseline. Among the male relatives, there was a statistically significant increase in antihypertensive medication uptake among those with hypertension (pre: 13%, *n* = 6/46; post: 17%, *n* = 8/46; *X*^2^: 32.7750, *p*-value: < 0.0001) relative to baseline.

**Conclusion:**

HIV aPS holds promise for integrated HIV and hypertension screening among at-risk clients and their families. Twenty-nine percent of the male relatives had hypertension, higher than the national prevalence (24%), while one-third had pre-hypertension. We observed relatively high participant retention, reductions in blood pressure, and increase in antihypertensive medication uptake among those with confirmed hypertension. Future research expanding the aPS model to other non-communicable diseases through larger studies with longer follow-ups is required to better assess causal relationships and optimize integrated service delivery.

## Contributions to the literature

- HIV assisted partner services (aPS) have been shown to be effective in HIV-case finding among sex partners to newly diagnosed HIV-positive individuals.
- Many African countries are dealing with the double burden of communicable and non-communicable diseases.
- We, therefore, assessed the feasibility of utilizing the aPS model to support integrated HIV and hypertension screening.
- The high prevalence of hypertension and prehypertension among male relatives to women diagnosed with hypertension receiving HIV testing services is of grave concern.
- The aPS model shows promise in providing family-based, integrated disease management.

## Background

African countries are increasingly facing the double burden of communicable and non-communicable diseases (NCDs), especially cardiovascular diseases (CVDs) [1]. According to the World Health Organization (WHO), CVDs are the leading cause of death globally with over 75% of these deaths occurring in low and middle-income countries, particularly in Africa [2]. Hypertension is the leading risk factor for CVDs [2]. However, despite 24% of all adults in Kenya having hypertension, only 16% of them are aware of their hypertensive status [3]. HIV infection is also known to be a risk factor in the development of hypertension through derangement of lipid metabolism and immune activation [4]. In Kenya, the HIV prevalence is estimated at 5% with approximately 80% of persons living with HIV (PLWH) aware of their HIV-positive status [5].

Due to disparities in HIV and hypertension awareness, offering hypertension screening to individuals receiving HIV testing service (HTS) can potentially promote early diagnosis of hypertension among individuals with or at risk of HIV infection. One intervention to improve HIV screening that can be leveraged is HIV assisted partner services (aPS) - where consenting PLWH are assisted by a trained healthcare provider to disclose their status or to anonymously notify their sexual and / or drug injecting partner(s) of their potential exposure to HIV [6]. Several clinical trials and programmatic evaluations conducted in Africa have demonstrated the effectiveness of aPS in increasing HIV testing and linkage to care [7–13].

Data from several programs in Kenya, including the Healthy Heart Africa (HHA) initiative indicate that men are less likely than women to be screened for hypertension (36% versus 63%) and even less likely to link to care if diagnosed [14, 15]. Similar observations have been made in HIV programs in Kenya; despite numerous interventions to improve HIV testing in accordance with the UNAIDS 95-95-95 targets, 73% of men in Kenya are aware of their HIV status compared to 83% of women [5]. By leveraging HTS infrastructure, facilities can be used to offer both HIV and hypertension screening services. We, therefore, evaluated the feasibility of the HIV aPS model in supporting integrated HIV and hypertension screening in Nairobi, Kenya.

## Methods

### Study design and setting

We conducted a pre-and post-intervention study between August 2019 and December 2020 at the Kenyatta National Teaching and Referral Hospital (KNH) Voluntary Counselling and Testing (VCT) Center. KNH is the largest public facility in Kenya and was selected due to its diverse population of clients living with or without HIV [16].

### Study participants

We recruited women with confirmed hypertension receiving HTS at KNH VCT who may or may not have been HIV-positive (female index clients). Eligible female index clients were at least 35 years of age, not pregnant, willing to provide informed consent and male relative information, and residing within 50 km of the study site. Male relatives, including husbands, sons, brothers, grandsons, fathers etc., were at least 35 years old and resided within 50 km of the study site.

### Intervention

Female index clients were interviewed by study staff to obtain information on their male relatives. Study staff were HTS providers i.e., Ministry of Health certified facility-based lay workers with diplomas in social science or counseling psychology and certificate training on HTS. Male relatives were then traced on phone or in person at least 4 times over a 1 month duration by study staff. Those successfully traced were informed of the study, offered hypertension screening and HIV testing at the nearest hospital, home, or convenient venue, and referred for management, as appropriate. Participants testing HIV positive were referred to a HIV comprehensive care clinic, while those with confirmed hypertension were referred to a physician for management.

At baseline and on 3 month follow-up, enrolled participants received counselling on dietary and lifestyle management. At 3-months, participants with hypertension were reviewed for blood pressure control while those known to be HIV positive were reviewed for ART initiation and viral suppression. Participants with either poorly controlled blood pressure or had not initiated ART at follow-up were counselled and referred to the medical outpatient clinic or to the CCC for management, respectively. We have included the template for intervention description and replication (TIDieR) recommendations checklist.

### Study procedures

Socio-demographic characteristics and anthropometric measurements (weight, height, waist, and hip circumference) were collected from enrolled participants at the baseline and 3-month follow-up visit. HIV infection was confirmed using rapid tests according to the national HTS protocol [17].

Blood pressure was measured using the automatic Omron M6 comfort (OMRON Healthcare, Japan) at three time points in the sitting position, 5 min apart. The Omron machines were calibrated daily following the manufacturer’s protocol to ensure accurate blood pressure readings. Hypertension was defined as a systolic blood pressure (SBP) ≥ 140 mm Hg and / or diastolic blood pressure (DBP) ≥ 90 mm Hg across three measurements on two occasions, and/or use of antihypertensive medication according to the Kenya guidelines [18]. Those with blood pressure levels above 140/90 mmHg were referred to the medical outpatient clinic for confirmation within 6 weeks before being confirmed as having hypertension. Pre-hypertension was defined as blood pressure of between 130/80 mmHg and 139/89 mmHg.

Weight was measured to the nearest ± 0.1 kg using ADE spring scale (1–150 kg). Height, waist, and hip circumferences were measured to the nearest ± 0.1 cm using a stadiometer SECA 213 portable stadiometer (SECA, German) or a mounted tape measure on a hard and level surface. Each week, the weight scale was calibrated using standard weights, while the height scale and mounted tape were calibrated using a standard one meter metallic rod. Body mass index (BMI) was calculated as weight/height^2^ (kg/m^2^) and the cut-off points were severely underweight < 16.5 kg/ m^2^, underweight: 16.5 to 18.5 kg/ m^2^, normal weight: 18.5 to 24.9 kg/ m^2^, overweight 25 to 29.9 kg/ m^2^, and obesity ≥ 30 kg/ m^2^ [18]. Waist-hip ratio (WHR) was calculated as waist circumference / hip circumference and the cut-off points were normal: male < 0.9, female < 0.85, and central obesity: male ≥ 0.9, female ≥ 0.85.

### Data collection

Data was collected by study staff using interviewer-administered questionnaires programmed on mobile phones using the Open Data Kit platform before being uploaded onto a central server [12, 19]. This technology platform was programmed with validation checks to reduce data entry errors and improve efficiency in data management.

### Data analysis

We described baseline sociodemographic characteristics and the prevalence of hypertension and HIV. We compared baseline to 3-month SBP, DBP, BMI, and WHR using paired sample t-tests, and baseline to 3-month antihypertensive medication uptake using Pearson’s chi-square test. Analysis was conducted using STATA version 17.

### Ethical approval

This study received ethical approval from the KNH Ethical and Scientific Review Committee (P568/08/2018). All interviewees provided written informed consent prior to enrolment. For illiterate participants, informed consent to participate was taken from legal guardians.

## Results

### Female index client characteristics

**Fig. 1:**
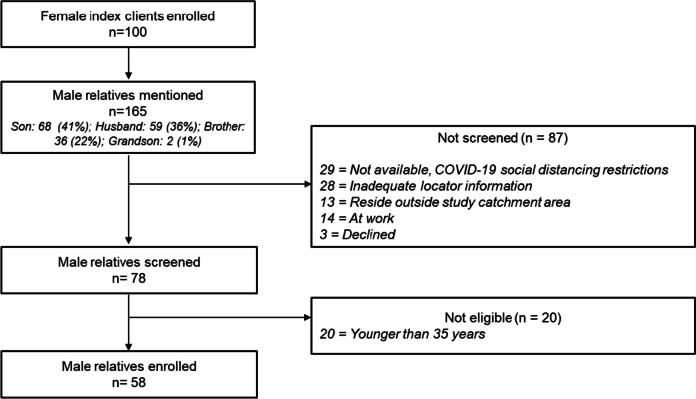
Flow diagram of participant screening and enrollment

We enrolled 100 female index clients who had a median age of 55 years (interquartile range, IQR: 47–65) (Fig. 1, Table 1). Two-thirds were in married monogamous relationships (66%, *n* = 66/100), and none were HIV-positive (0%, *n* = 0/100). The median BMI and WHR were 29 kg/m^2^ and 0.86, respectively.

**Table 1:** Baseline demographic characteristics

**Variable**	**Details**	**Female index clients** ***n* = 100** n (%), median (IQR)	**Overall male relatives** ***n* = 58** n (%), median (IQR)	Husbands *n* = 27 n (%), median (IQR)	Sons *n* = 18 n (%), median (IQR)	Brothers *n* = 12 n (%), median (IQR)	Grandson *n* = 1 n (%), median (IQR)
**Age**	Years	55 (47–65)	49 (40–59)	56 (46–68)	43 (39–51)	49 (40–51)	35 (35–35)
**Education**	Primary	62 (62%)	19 (33%)	12 (44%)	3 (17%)	4 (33%)	-
	Secondary	34 (34%)	29 (50%)	12 (44%)	10 (56%)	6 (50%)	1 (100%)
	Tertiary	3 (3%)	10 (17%)	3 (11%)	5 (27%)	2 (17%)	-
	None	1 (1%)	-	-	-	-	-
**Marital status**	Married monogamous	66 (66%)	53 (91%)	26 (96%)	17 (94%)	10 (83%)	-
	Widow / widower	20 (20%)	1 (1%)	1 (4%)	-	-	-
	Divorced / separated	9 (9%)	1 (1%)	-	-	1 (8%)	-
	Single	5 (5%)	2 (3%)	-	-	1 (8%)	1 (100%)
	Married polygamous	-	1 (1%)	-	1 (6%)	-	-
**HIV status**	Positive	-	-	-	-	-	-
	Negative	96 (96%)	55 (95%)	26 (96%)	16 (89%)	12 (100%)	1 (100%)
	Declined testing	4 (4%)	3 (5%)	1 (4%)	2 (11%)	-	-
**Hypertension status**	Hypertension	100 (100%)	17 (29%)	8 (30%)	7 (39%)	2 (17%)	-
	Pre-hypertension	-	20 (34%)	10 (37%)	6 (33%)	4 (33%)	-
**Blood pressure**	Median mmHg	154/95 (145/90–167/100)	127/79 (120/76–139/85)	130/79 (120/76–140/84)	130/80 (125/77–140/90)	120/77 (118/76–131/85)	119/70 (119/70–119/70)
**Body mass index**	Kg/m^2^	29 (25–33)	23 (22–26)	24 (23–26)	24 (22–26)	23 (22–25)	20 (20–20)
**Waist/hip ratio**	Ratio	0.86 (0.83–0.90)	0.92 (0.87–0.95)	0.94 (0.87–0.97)	0.92 (0.89–0.95)	0.87 (0.86–0.89)	0.94 (0.94–0.94)

*IQR* Interquartile range, *Hypertension* Defined as systolic blood pressure ≥ 140 mm Hg and / or diastolic blood pressure ≥ 90 mm Hg and / or use of antihypertensive medication, *Pre-hypertension* Defined as blood pressure of between 130/80 mmHg and 139/89 mmHg. Of the 100 female index clients with hypertension, 92 (92%)were on antihypertensive medication. Of the 58 male relatives, 9 (16%) were on antihypertensive medications - all of whom had confirmed hypertension

### Male relative characteristics

The female index clients mentioned 165 male relatives namely, brothers: 68 (41%), husbands: 59 (36%), sons: 36 (22%), and grandsons: 2 (1%) (Fig. 1). Of these, 35% (*n* = 58/165) were enrolled into the study. Main reasons for low male relative enrollment were COVID-19 social distancing restrictions at the time of the study, inadequate locator information, residing outside the study catchment area, and being younger than 35 years.

The 58 enrolled male relatives had a median age of 49 years (IQR: 40–59), 91% were in married monogamous relationships (*n* = 53/58), and none (0%, *n* = 0/58) were HIV positive (Table 1). About one-third of the male relative had pre-hypertension (34%, *n* = 20/58), while 29% had hypertension (*n* = 17/58) i.e., husbands (30%, *n* = 8/27), sons 39%, *n* = 7/18), brothers (17%, *n* = 2/12), grandsons (0%, *n* = 0/1). The median BMI and WHR were 23 kg/m^2^ and 0.92, respectively.

### Female index client retention at the 3 month visit

Of the 100 enrolled female index clients, 92 (92%) returned for the 3-month visit. Of the eight who were lost to follow-up, the reasons included being out of town (63%, *n* = 5/8) and death (38%, *n* = 3/8). The three deaths, recorded from the death certificates presented by the relatives, occurred after hospitalization due to renal failure (*n* = 2) and septic shock after eye surgery in a client with hypertension and diabetes (*n* = 1). All deaths were reported to the KNH Ethical and Scientific Review Committee as per the study protocol.

### Male relative retention at the 3-month visit

**Fig. 2:**
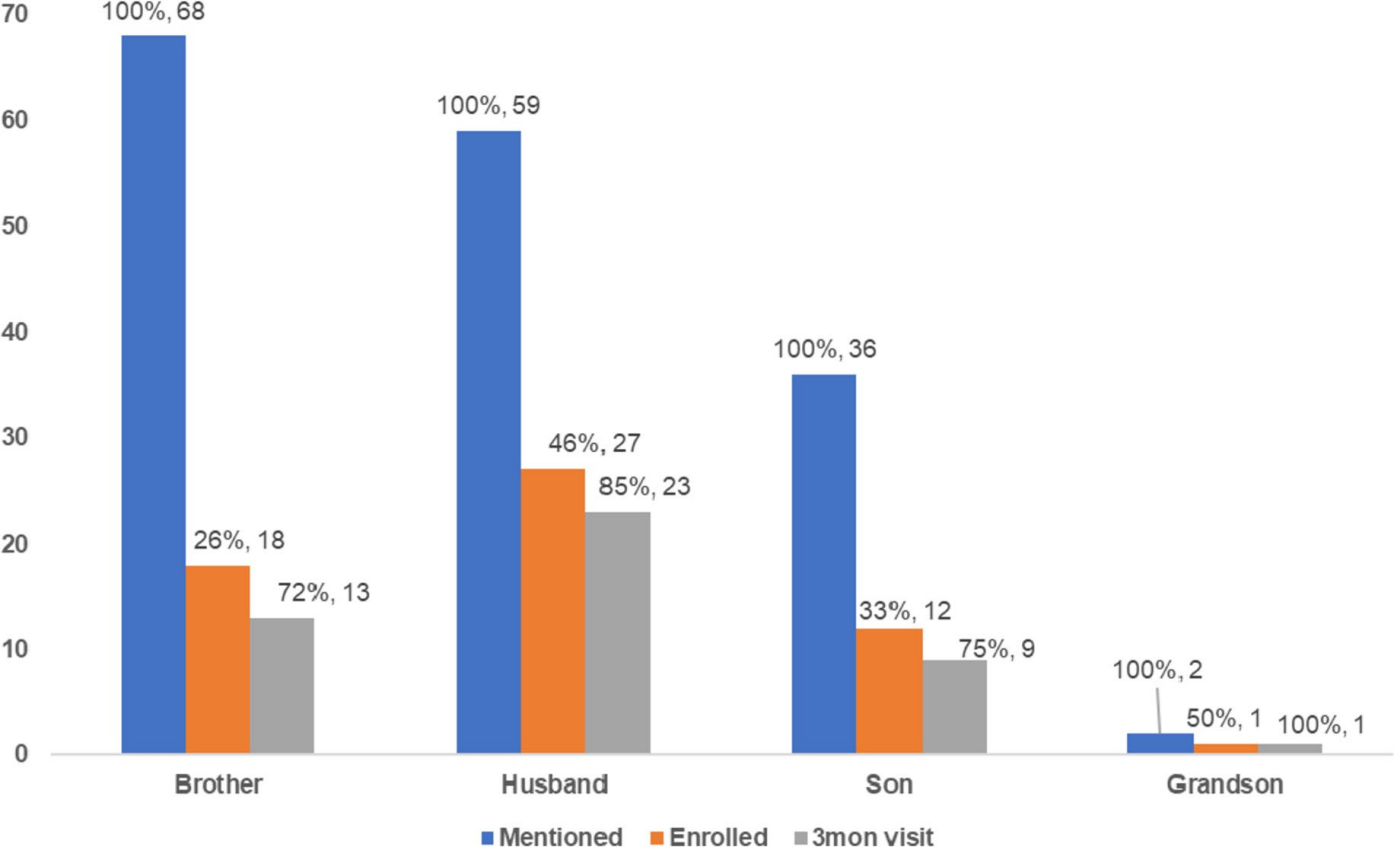
Male relatives mentioned, enrolled, and retained at the 3-month visit

Of the 58 enrolled male relatives, 46 (79%) returned for the 3-month visit. Of the 12 who were lost to follow-up, the reasons included unreachability on phone and / or in-person (83%, *n* = 10/12), being out of town (8%, *n* = 1/12), and unavailability for clinic visit due to work (8%, *n* = 1/12). Less than half of the mentioned male relatives were enrolled in each category (husband: 46%, *n* = 27/59; son: 33%, *n* = 12/36; brother: 26%, *n* = 18/65; grandson: 50%, *n* = 1/2) (Fig. 2). After enrolment, retention among enrolled male relatives was relatively high across the categories (husband: 85%, *n* = 23/27; son: 75%, *n* = 9/12; brother: 72%, *n* = 13/18; grandson: 100%, *n* = 1/1).

### Changes in anthropometric measures and antihypertensive medication uptake

Among the 92 female index clients at the 3-month visit, there was a statistically significant decline in post-intervention SBP and DBP, and increase in antihypertensive medication uptake, relative to baseline (Tables 2 and 3). However, there was no difference between the baseline and 3-month BMI and WHR.

**Table 2:** Changes in anthropometric measures between baseline and 3-month follow-up

	**Baseline visit**	**3-month visit**	**Mean difference**	**Lower 95% CI**	**Upper 95% CI**	***P*-value**
**Female index clients (*n* = 92)**						
Systolic BP	156	133	23	19	26	< 0.0001
Diastolic BP	97	80	17	14	19	< 0.0001
Body mass index	29	29	0.1	-0.4	0.5	0.7317
Waist / hip ratio	0.88	0.86	0.02	-0.01	0.05	0.2569
**Male relatives (*n* = 46)**						
Systolic BP	129	127	3	-2	8	0.2697
Diastolic BP	81	78	2	-2	6	0.2649
Body mass index	24	24	-0.1	-0.2	0.1	0.3936
Waist / hip ratio	0.91	0.91	0	-0.01	0.01	0.6341

*CI* Confidence interval, *BP* Blood pressure

**Table 3:** Changes in antihypertensive medication uptake between baseline and 3-month follow-up

	**Baseline visit**	**3-month visit**	***X***^**2**^	***P*-value**
**Female index clients (*n* = 92)**				
**Hypertension status**				
**On anti-hypertensive medication**				
**Overall**	**84 (91%)**	**90 (98%)**	**4.3931**	**0.036**
Hypertensive	84 (91%)	90 (98%)		
Pre-hypertensive	0 (0%)	0 (0%)		
Normal BP	0 (0%)	0 (0%)		
**Not on anti-hypertensive medication**				
**Overall**	**8 (9%)**	**2 (1%)**		
Hypertensive	8 (9%)	1 (1%)		
Pre-hypertensive	0 (0%)	1 (1%)		
Normal BP	0 (0%)	0 (0%)		
**Male relatives (*n* = 46)**				
**On anti-hypertensive medication**				
**Overall**	**6 (13%)**	**8 (17%)**	**32.7750**	**< 0.0001**
Hypertensive	6 (13%)	8 (17%)		
Pre-hypertensive	0 (0%)	0 (0%)		
Normal BP	0 (0%)	0 (0%)		
**Not on anti-hypertensive medication**				
**Overall**	**40 (87%)**	**38 (83%)**		
Hypertensive	5 (11%)	2 (4%)		
Pre-hypertensive	18 (39%)	16 (35%)		
Normal BP	17 (37%)	20 (43%)		

*X*^*2*^ Pearson chi-square statistic

Among the 46 male relatives at the 3-month visit, there was a statistically significant increase in post-intervention antihypertensive medication uptake among those with hypertension relative to baseline (Table 3). However, there was no statistically significant difference between pre- and post-intervention SBP, DBP, BMI, and WHR.

## Discussion

In this study utilizing the aPS model to support integrated HIV and hypertension screening, female index clients mentioned not only their husbands, but also their brothers, sons, and grandsons, widening the reach to men who may otherwise have never received HIV and hypertension screening services. Despite low enrolment among the male relatives, 29% of those enrolled had hypertension, 34% had pre-hypertension, and none were HIV-positive. At the 3 month follow-up visit, we observed relatively high retention among participants with statistically significant increase in antihypertensive medication uptake among those with hypertension. We also observed significant reductions in SBP and DBP among female index clients, though no significant changes in blood pressure were observed among enrolled male relatives.

To our knowledge, this is the first study in Kenya showing spousal and maternal associations in hypertension risk, highlighting the potential of the aPS model to support targeted hypertension screening among families with shared genetic and lifestyle related risk factors. In a longitudinal cohort study evaluating spousal metabolic risk factors and incident hypertension in Iran, there was increased risk of hypertension from having a spouse with diabetes mellitus [20]. Although they did not evaluate the children of such couples, the study highlighted the potential of using NCD health data from one family member to evaluate risk and guide the screening of the other. Interestingly, no fathers to the female index clients were mentioned in our study, either because they were deceased or resided outside of study catchment area, indicating a missed opportunity for screening. Fathers, as well as other older relatives, are at a higher risk for hypertension and other age-related morbidities. They, together with their families, may benefit from targeted screening for HIV and hypertension among other NCDs.

Overall, there was low enrolment of male relatives due to COVID-19 social distancing restrictions which was largely unavoidable [21]. However, a significant proportion of those not enrolled had inadequate locator information which was surprising as these were close relatives to the female index clients. Inadequate locator information e.g., wrong phone numbers, residential / workplace addresses, names, has been a challenge in previous aPS studies [22, 23]. Healthcare providers offering aPS will require training and support in creating rapport and building trust with index clients as they elicit contact information.

Twenty-nine percent of the enrolled male relatives had hypertension, higher than the national hypertension prevalence in Kenya (24%) [3], while 34% of them had pre-hypertension. This is quite alarming and indicates the value of targeted hypertension screening among family members to individuals known to have hypertension. A similar approach may be used for other NCDs that are common among families, e.g., diabetes mellitus - whose risk factors include central obesity which we observed among our participants. In Tanzania, the prevalence of diabetes mellitus and renal failure among hypertensive PLWH was 9% and 29%, respectively, while 53% had an intermediate to high 10-year risk of an atherosclerotic cardiovascular disease (ASCVD) event [24], flagging the risk for comorbidities in this population group. As PLWH grow older, policymakers will need to integrate NCD management into HIV programs to avert such complications. This is all the more important given Kenya’s concerns over low HIV testing yields – defined as proportion of individuals newly testing HIV-positive out of total individuals tested for HIV. Kenya has a low national HIV testing yield of approximately ~1% similar to our study where none of the participants were HIV-positive [25]. The available HIV infrastructure can, therefore, be more effectively utilized to support integrated service delivery for both communicable and NCDs.

There was relatively high participant retention and anti-hypertensive medication uptake at 3-months, potentially due to the family-centered approach to screening. In our qualitative study, participants preferred such hypertension screening models due to the inbuilt family support systems [26]. Policymakers may need to adopt integrated service delivery models to PLWH with NCD related comorbidities [27]. One such example is multimonth dispensing of ART and antihypertensive medications which was shown to improve hypertension control, viral suppression, and retention to care among hypertensive PLWH in Uganda [28]. We also observed changes in anthropometric measures at the 3-month visit with statistically significant declines in blood pressure among female index clients, though this was not observed among the male relatives. In a pilot study in Tanzania, researchers observed similar blood pressure declines over a 4-week period when utilizing a community healthcare worker delivered educational intervention to support integrated hypertension care engagement in HIV programs [29]. Such people-centered care models hold promise in improving care and management of hypertension, and potentially other NCDs, within HIV care programs.

Our study had several strengths. First, we evaluated the feasibility of the aPS model in supporting integrated HIV and hypertension screening services, contributing to literature on its potential. Second, this study was conducted at KNH, the largest teaching and referral hospital in Kenya that receives patients from the entire country, improving the generalizability of study results. Third, our study design included a pre-post intervention assessment of our outcomes of interest allowing us to assess changes over time.

Among the limitations, we had a small sample size due to COVID-19 social distancing restrictions that limited participant access. Second, we had a 3-month follow-up duration that may not be sufficient in evaluating long-term blood pressure control and lifestyle management. Third, our study design lacked both random assignment and a control group. It is, therefore, challenging to assess whether the direction and magnitude of the changes would have been different from a placebo group or if they were due to natural maturation. We, however, believe that the findings from our study provide insights to integrated HIV and hypertension screening services.

## Conclusion

The HIV aPS model holds promise in supporting targeted HIV and hypertension screening among at-risk clients and their families. This model can also be expanded to support widespread screening services for other non-communicable diseases e.g., targeted diabetes screening of at-risk relatives to individuals diagnosed with diabetes. Future research with larger studies involving longer follow-ups is required to better assess causal relationships and optimize integrated service delivery models.

## Data Availability

The datasets used and/or analyzed during the current study are available from the corresponding author on reasonable request.

## Abbreviations

aPS: Assisted partner services
BMI: Body mass index
CVD: Cardiovascular disease
DBP: Diastolic blood pressure
HTS: HIV testing service
IPV: Intimate partner violence
IQR: Interquartile range
KNH: Kenyatta National Teaching and Referral Hospital
MOH: Ministry of Health
NCD: Non-communicable disease
PLWH: People living with HIV
SBP: Systolic blood pressure
TIDieR: Template for intervention description and replication
VCT: Voluntary counselling and testing
WHO: World Health Organization
WHR: Waist hip ratio
